# A sharing practices review of the visual search and eye movements literature reveals recommendations for our field and others

**DOI:** 10.3758/s13428-025-02759-3

**Published:** 2025-07-25

**Authors:** Hayward J. Godwin, Haden Dewis, Peter T. Darch, Michael C. Hout, Daniel Ernst, Philippa Broadbent, Megan Papesh, Jeremy M. Wolfe

**Affiliations:** 1 School of Psychology, Highfield, University of Southampton, Southampton, Hampshire SO17 1BJ UK; 2 University of Illinois at Urbana-Champaign, Urbana, IL USA; 3 New Mexico State University, Las Cruces, NM USA; 4 Bielefeld University, Bielefeld, Germany; 5 University of Massachusetts Lowell, Lowell, MA USA; 6 Visual Attention Lab, Brigham and Women’s Hospital, Boston, MA USA; 7 Harvard Medical School, Boston, MA USA

**Keywords:** Visual search, Eye tracking, Data sharing, Secondary data analyses, Open science, Sharing practices review

## Abstract

The sharing of research outputs is an important endeavor, one that is increasingly required by funders and publishers alike. Here, we catalogued and examined data sharing practices, using our own field of visual search and eye movement behavior as an example. To find outputs from scientific research, we conducted two searches: a Literature Search and a repository search. Overall, we found that researchers in our field generally shared outputs that enabled others to analytically reproduce published results. It was rare for researchers to share outputs that enabled direct replications of their work, and it was also rare for researchers to share raw data that would enable secondary data analyses. Comparing the results of our two searches of the literature, we found that a lack of metadata substantially reduced the rates at which outputs could be found and used. Based on our findings, we present a set of recommendations summarized in our ‘Find It – Access It – Reuse It’ scorecard. The scorecard is intended to assist researchers in sharing outputs in a manner that will enable others to better find, access, and understand them – and this includes researchers in other fields beyond our own.

## Introduction

In recent decades, key research stakeholders, including governments, funding agencies, disciplinary societies, universities, and journals, have promoted the sharing of datasets produced from research. Borgman (2017) found that the four arguments most commonly advanced by policy-makers to support data sharing were:1) the ability to ask new questions using extant data, thereby increasing the return on investments in data collection; 2) enhanced transparency and reproducibility; 3) advancing the state of scholarship, for instance through enabling the building of data collections; and 4) enhancing the rights of the public to access taxpayer-funded datasets. Motivated by these rationales, stakeholders have taken different approaches to promote data sharing. Funding agencies now require data management plans (European Union Publications Office, 2018; National Science Foundation, 2010), while many journals require authors to make supporting datasets openly available (Stodden, 2020). Other stakeholders have invested in infrastructure to support data sharing and reuse, such as digital repositories and metadata schema (Rauch et al., 2022; Yakel et al., 2024). Given the proliferation of policies and investments, it is essential to evaluate the extent to which data sharing and reuse goals have been achieved. The current project addresses this issue by utilizing research on visual search and eye movements as a case study. Based on our findings, we present recommendations for researchers seeking to enhance their research outputs, not just in our field but in other fields as well.

As a discipline, psychology has received an especially high level of attention regarding sharing practices. One key motivation arises from a project that sought to replicate the results of multiple prior experiments published in high-profile journals (Open Science Collaboration, 2015). Many of these attempted replications failed. Combined with other high-profile controversies in Psychology (see Korbmacher et al., 2023), this has been described as a ‘replication crisis’ (Open Science Collaboration, 2015) that has received significant media coverage (Baker, 2016). The sharing of scientific outputs, particularly datasets, has been touted as a potential measure to safeguard the field against future crises (Nosek & Bar-Anan, 2012).

Different approaches have been taken regarding the characterization of researchers’ data-sharing. For example, the FAIR principles (Wilkinson et al., 2016) recommend that digital research outputs be*: Findable* and *Accessible* by prospective reusers (here, a ‘reuser’ is an individual seeking to use an extant dataset for another purpose); *Interoperable* with systems used by prospective reusers; and *Reusable* by being accompanied by information explaining its contents and provenance. Decisions made about what to include in the shared dataset and what additional materials, such as metadata, to share alongside the dataset determine if and how the dataset can be reused. For instance, computational reproducibility requires supplying relevant code along with raw data (Stodden et al., 2013). Of course, not all datasets can be shared openly, for instance, to safeguard human subjects’ privacy, mitigate security risks, or protect commercial interests.

The FAIR principles were initially formulated to promote automated dataset discovery and reuse, meaning that the recommendations for implementing them often have little relevance to many researchers (Henriksen & Mundt, 2024). Moreover, they have been criticized for being too general, leading to disparate applications by researchers (Candela et al., 2024). Nevertheless, these principles have proven useful as a broad heuristic for evaluating the manual processes of searching, retrieving, and evaluating datasets that align with research practices in fields such as psychology (Bishop & Collier, 2022).

A number of researchers have conducted metascience projects that have catalogued in detail what has been shared alongside publications. For example, Hardwicke et al. (2020) examined 250 publications from the social sciences literature and found that it was rare for researchers to share raw data, materials from experiments, and analytic scripts. This was followed up by Hardwicke et al. (2022), where the authors examined 250 publications from the psychology literature only, and again found that the sharing of raw data, materials, and analytic scripts was rare. Most recently, Hardwicke et al. (2024) examined 400 publications from psychology papers published in the year 2022, and found that, despite all of the efforts to improve sharing of scientific outputs, that sharing practices had only increased by a small degree compared to their earlier examinations of the literature (see also Hardwicke & Ioannidis, 2018).

Several researchers have conducted projects that have catalogued in detail what has been shared alongside publications. For example, Hardwicke et al. (2020) examined 250 publications from the social sciences literature and found that it was rare for researchers to share raw data, materials from experiments, or analytic scripts. This was followed up by Hardwicke et al. (2022), where the authors examined 250 publications from the psychology literature only, and again found that the sharing of raw data, materials, and analytic scripts was rare. Most recently, Hardwicke et al. (2024) examined 400 publications from psychology papers published in the year 2022, and found that -- despite all of the efforts to improve sharing of scientific outputs -- sharing practices had only increased by a small degree compared to their earlier examinations of the literature (see also Hardwicke & Ioannidis, 2018).

A different approach to understanding sharing practices is to engage in a quality appraisal of the outputs shared, adopting a systematic review approach. Towse et al. (2021) recently conducted just such an examination of the experimental psychology literature. Following an approach developed by Roche et al. (2015), Towse et al. (2021) found that 51% of datasets linked to a sample of articles from mainstream psychology journals were incomplete (i.e., not all data, including raw data were shared), and 68% were not reusable (i.e., not all data could be reused and understood by others). The primary barriers to reusability involved a lack of sharing raw data, and a lack of metadata describing the datasets that were shared. Metadata can take many forms when it comes to scientific outputs. Here, the key metadata required involves guidance notes, lists, tables, and other information that enables other researchers to understand what outputs have been shared, and what they can be used for. For example, when datasets are shared, the most valuable form of accompanying metadata is a guide or ‘data dictionary’ that provides guidance and information regarding what information is contained within the different columns and rows of the dataset. Without that information, other researchers are left to engage in (educated) guesses regarding what the values in the shared dataset mean.

The projects described above employed a general approach, examining a wide array of different subfields across multiple journals. More recently, it has become common for researchers to focus on specific subfields or journals (see Rochios & Richmond, 2024). Although doing so may reduce the generality of any conclusions that can be drawn, the benefit of taking such an approach is that researchers can tailor their analyses and conclusions to the idiosyncrasies of their specific sub-fields. For example, Huff and Bongartz (2023) found that data sharing in educational psychology publications was very rare, and only increased by a small degree between 2018 and 2020. Meanwhile, Hardwicke et al. (2018) found that sharing increased following a requirement to share outputs implemented at the journal *Cognition*, a finding echoed by a study of the effects of a similar policy by the *Journal of Memory and Language* (Laurinavichyute et al., 2022). Crüwell et al. (2023) examined the benefits of introducing open data badges at the journal *Psychological Science,* finding that the badges increased rates of data sharing, but not to the extent of facilitating reproducibility*.* More recently, Wiechert et al. (2024) examined publications in the false memory literature, cataloguing 388 articles published between 2015 and 2023, finding that sharing practices improved during that time.

Here, we built upon the growing body of work that focuses on specific subfields and examined the outputs of publications relating to visual search and eye movement behavior. We conducted what we refer to as a *sharing practices review*, inspired by the FAIR principles and by previous research in this area, customized to suit the idiosyncrasies of research and outputs within our field. Datasets derived from visual search and eye movement experiments are excellent candidates for sharing and reuse because they are expensive and time-consuming to collect, and because the data can be analyzed in many different ways to produce new scientific knowledge (for a review, see Godwin et al., 2021).

The purpose of our sharing practices review was to catalogue the sharing of outputs by researchers in our field. To achieve this, we collated information from two different searches: we performed a *literature search*, using the Web of Science and a *repository search*, searching the Open Science Framework’s online repository for shared outputs (see methods, below). We then examined the outputs shared from publications that were found via each of the searches. Our examination of the outputs was conducted to determine what forms of reuse those outputs might facilitate. First, we addressed whether the outputs facilitated *analytic reproducibility*, i.e., reproducing statistical analyses that were presented in a publication. Second, we addressed whether the outputs could facilitate *direct replications* by sharing digital objects such as the software used to run experiments and/or materials such as stimuli used in the experiments. Third, we addressed whether the outputs enabled *secondary data analyses* to take place. This would involve the sharing of raw data that would enable the raw data to be processed and analyzed to address new research questions. Finally, in a step that is novel to this project, we cross-compared the literature search and repository search, and to prelude, found some important.

Aside from cataloguing how different forms of reuse were supported by outputs from publications in our subfield, our searches offer the further benefit of amassing a substantial database of available scientific outputs that will be of value to others in this field, thereby furthering one of the goals of data sharing identified by Borgman (2017); namely “advancing the state of scholarship.”

## Method

### Approach

We conducted two searches, looking for outputs of experiments involving visual search and eye tracking. First, we conducted a *literature search*, which involved examining individual publications to determine whether they shared data, code, or materials. Then, we conducted a *repository search*, which involved searching online repositories. Because the literature search revealed that researchers overwhelmingly used the Open Science Framework’s repositories to share data (OSF: https://osf.io), our repository search used the OSF to find and catalogue available datasets and other shared outputs.

### Open research practices

All of our analytic code, data, and results are available publicly at https://osf.io/5tmey/. Searches were not pre-registered.

### Search strategy

In both searches, we employed key search terms, and variants thereof, related to visual search and eye-tracking.

#### Literature search

We used Web of Science to identify potentially relevant publications that could contain datasets from visual search and eye-tracking experiments. We searched titles and abstracts for words relating both to visual search and eye tracking. A wildcard character (*) was added to the end of these words to find combinations with other terms. Our final query was:

(TI=(‘eye*’ OR ‘fixation*’ OR ‘saccade*’ OR ‘gaze*’) AND TI=(‘search’ or ‘visual search’ or ‘visual-search’ OR ‘categorical search’ OR ‘hybrid search’ OR ‘contextual cueing’ OR ‘scene search’)) OR (AB=(‘eye*’ OR ‘eye*’ OR ‘fixation*’ OR ‘saccade*’ OR ‘gaze*’) AND AB=(‘search’ or ‘visual search’ or ‘visual-search’ OR ‘categorical search’ OR ‘hybrid search’ OR ‘contextual cueing’ OR ‘scene search’))

We conducted our initial search on June 14, 2023, returning 16,224 results. We then filtered the results to focus on those results that held the most promise for our purposes of finding available visual search and eye movement datasets.

#### Repository search

Our repository search involved the same basic search as the literature search, except that it used the OSF’s search engine rather than the Web of Science to create an initial list of matches. We queried the OSF’s Application Programming Interface (API) and downloaded the results using custom scripts written in R. We adapted our search query to fit the syntax requirements of the OSF’s API. The final query we used June 23, 2023, was:

(“search” OR “visual search” OR “visual-search” OR “categorical search” OR “hybrid search” OR “scene search” OR “contextual cueing”) AND (eye* OR fixation* OR saccade* OR gaze*)

The OSF repository has different categories of outputs (e.g., projects, files, and so on). Here, we conducted our search query on projects only, since these are the closest analogues to the publication-level search conducted as part of the literature search.

### Inclusion and exclusion criteria

#### Literature search

We limited the search to mainstream psychological journals with papers published in a six-year window from the start of 2017 to the end of 2022. Our decision to restrict the range of journals searched was inspired by the approaches of prior studies, which aimed to avoid a high rate of irrelevant results that do not report on standard visual search experiments (Hardwicke et al., 2018, 2021; Roche et al., 2015; Towse et al., 2021). The journals that we searched were:

*Attention, Perception & Psychophysics; Cognition; Cognitive Research: Principles and Implications; Cognitive Science; Current Biology; Frontiers in Psychology; Journal of Experimental Psychology: Human Perception and Performance; Journal of Eye Movement Research; PEERJ; Psychonomic Bulletin & Review; Perception; Psychological Science; Journal of Vision; PLOS One; Quarterly Journal of Experimental Psychology; Scientific Reports; Vision Research; and Visual Cognition.*

Articles were included in our sample if they:

- Presented primary research using human participants,
- Utilized a visual search task, and
- Recorded the eye movement behavior of participants.

We defined a visual search task as one wherein participants looked for a pre-defined target (or category of target) in a task requiring eye movements to find the target. We screened titles and abstracts for relevance, and any uncertainties were resolved by reading the full publication.

#### Repository search

We matched our inclusion and exclusion criteria with the literature search as closely as possible. However, our repository search did not focus on a subset of specific journals since there is no way to implement such a filter using the OSF’s search (i.e., because projects on the OSF, which are not necessarily part of publications, do not ask for a journal name when being created).

**Fig. 1:**
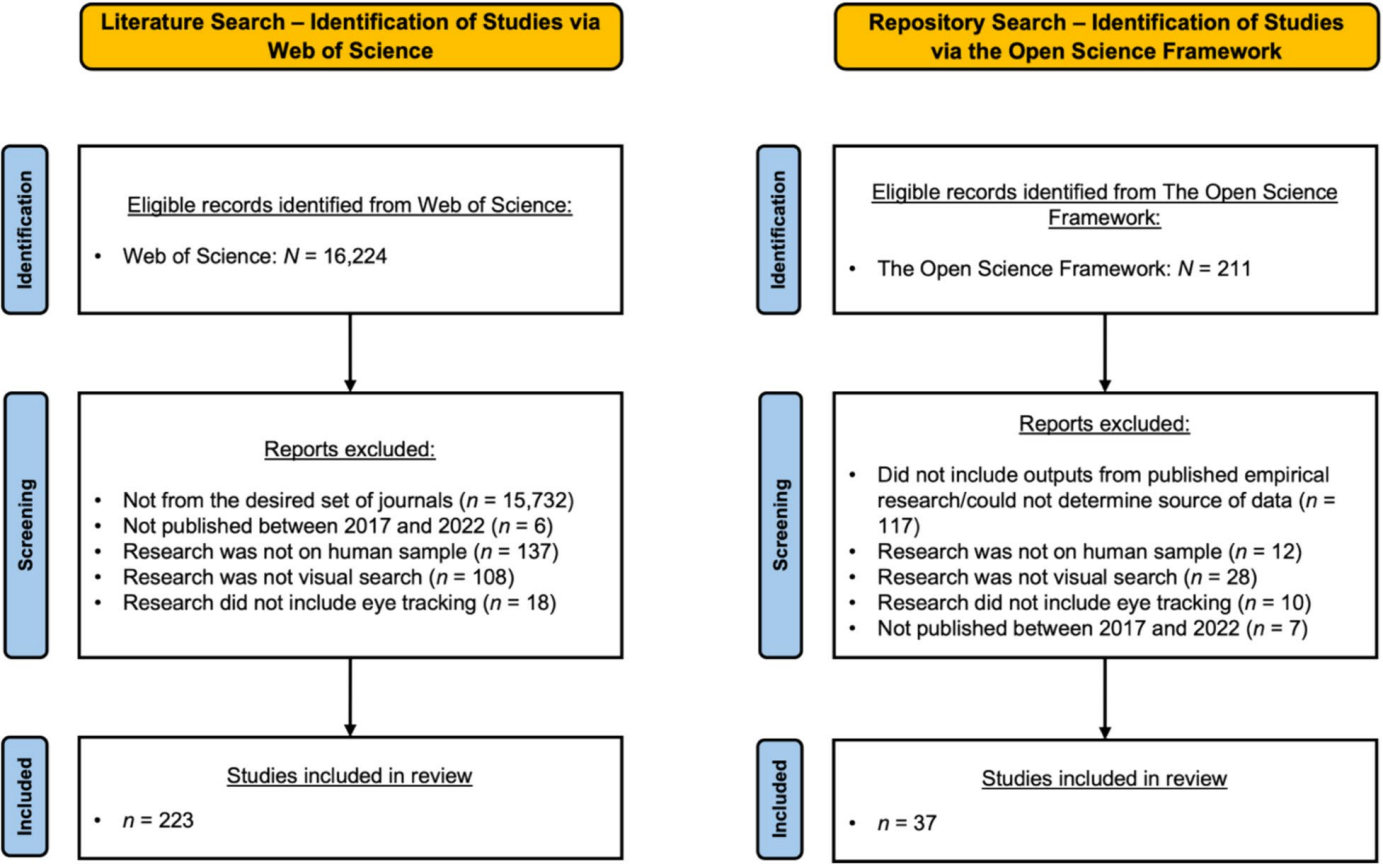
Selection and filtering process for the results of our two searches

The selection processes for both searches are summarized in Fig. 1.

#### Literature search

From our initial set of 16,224 results, we excluded publications based on the above criteria. Focusing only on specific journals reduced our set of publications to 492. Of these, six were removed because they were not in the specified year range. Next, we removed a further 137 publications that did not involve primary human subjects’ research. A total of 108 publications were then removed because they did not involve a form of visual search that met our inclusion criteria. Of the remaining 241 publications, 223 involved a combination of visual search and eye tracking.

#### Repository search

Our initial search yielded 211 results. We then screened these results against our criteria. We removed any OSF projects that did not include outputs from published empirical research or that included outputs for which we could not determine the source due to limited or absent metadata, a total of 117 projects. Next, we removed 12 projects that did not involve primary human subjects’ research. We then removed 28 projects that were not derived from visual search experiments, and a further ten projects that did not record participants’ eye movement behavior. Finally, we removed seven projects that fell out of the date range (2017–2022). A total of 37 projects remained for detailed examination.

### Data extraction

When reviewing the data sharing practices of the results returned by our searches, we catalogued them primarily in terms of the different forms of reproducibility that they can support. We began by determining both the overall rate of sharing within the sample and the rate at which links to outputs resulted in us being able to access those outputs.

We then examined what was shared by researchers to determine what types of reproducibility those outputs could support. This involved first checking whether the outputs supported analytic reproducibility (i.e., repeating or recreating the analyses presented in the publication). Next, we checked whether the outputs could support a direct replication by sharing materials (images, sounds, videos) and experimental software code. We then checked whether the outputs could enable secondary data analyses to take place – that is, whether they shared raw data that would be amenable to answering a wide range of new research questions.

Throughout our examinations of the types of reproducibility that the outputs could support, we also assessed the file formats that researchers used when sharing their outputs. For files to be classed as interoperable, they need to be provided in an open format, such as a text file, or in a format that can be used by freely available open-source software.

After assessing the outputs in terms of the different forms of reproducibility that they could support, we examined the metadata made available by researchers. We did this in two ways: first, by checking for the presence of guides, codebooks, or data dictionaries within the outputs shared. After this, we conducted a cross-comparison of the findings of both the literature search and repository search, which tapped into the metadata shared by researchers.

## Results

### Rates of sharing

We began our examination of the outputs by focusing on the overall rates of sharing. This only occurred for the outputs identified by our literature search, as all outputs were already available when discovered through our repository search.

Of the 223 publications included in our literature search, 78 (~35%) claimed to have shared some form of outputs from their work. Despite links to outputs being presented in publications, those links do not always result in the intended outputs being reachable (e.g., see Federer, 2022). For many reasons, websites can become inactive, or links can be provided to outputs that require a login or privileged access. In our literature search, outputs were accessible in ~90 % (*N* = 70) of the sample. Of the remaining ~10 % (*N* = 8), web links to the locations of these outputs did not allow access, for several reasons: web links were no longer accessible (two publications), zipped files which could not be opened (one publication), and incorrect links to OSF projects were provided (five publications). For this final category of incorrect links, these links to OSF projects were incorrect because publications’ authors copied and pasted the address of their OSF projects into the publications themselves. Doing so is not a problem for OSF projects that are publicly shared. However, in these five cases, those projects were still listed as ‘private’. As such, clicking on them leads to an OSF page that a visitor can use to request administrative powers over a project. If exploited with malicious intent, this could result in the data being altered, deleted, or otherwise changed by others without the knowledge of the project’s true owners. Because the outputs for our repository search had already been publicly shared online, there were no barriers to accessing them.

**Fig. 2:**
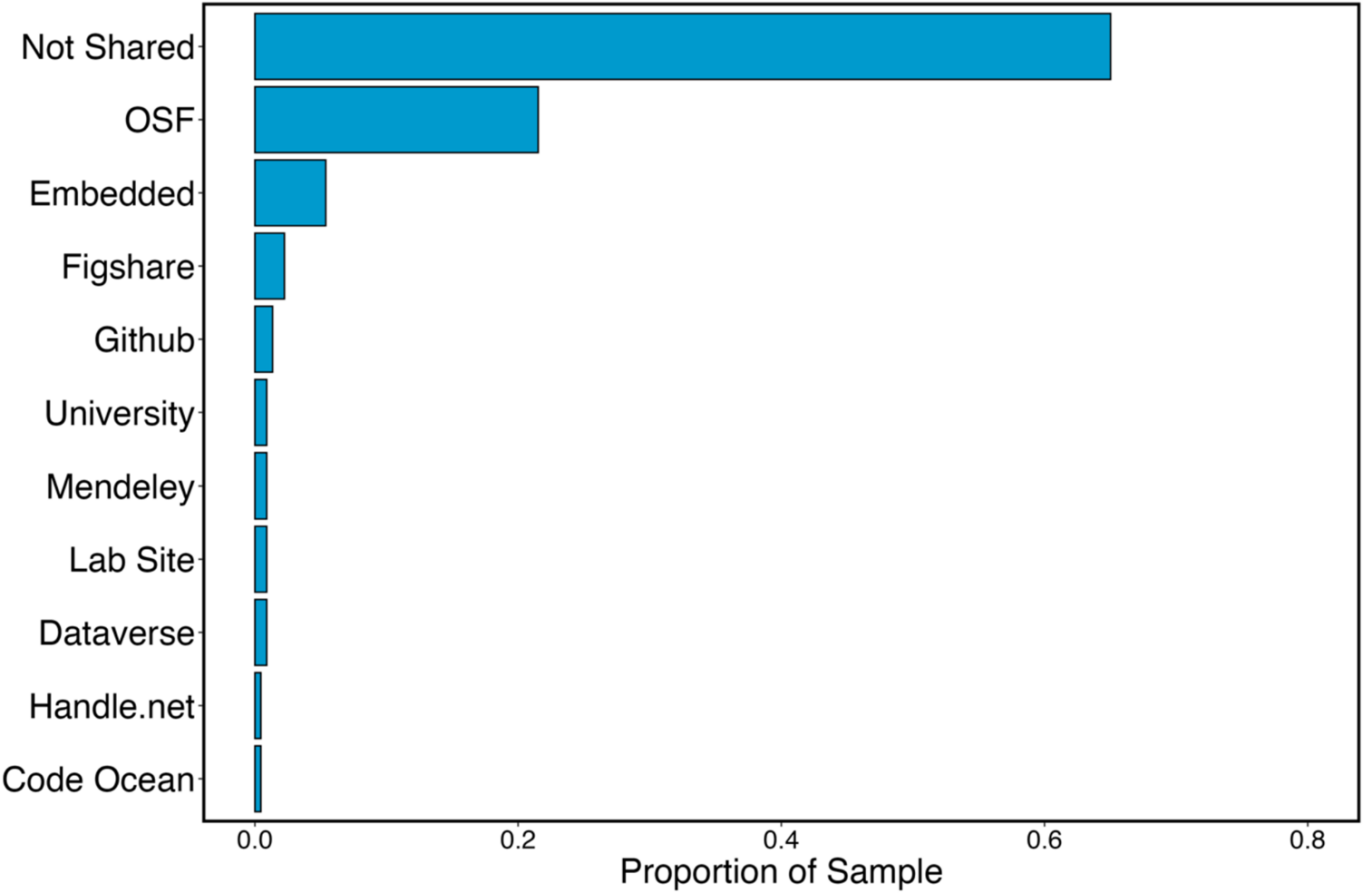
Literature search: Where were the outputs shared online?

Finally, we examined the websites that researchers used to share their outputs from publications found during our literature search (Fig. 2). The most popular sharing location (after sharing no outputs at all) was the Open Science Framework’s website (https://osf.io), used 21.52% (*N* = 48) of cases from our literature search. Indeed, this location was so popular that the next-most popular method (embedding links to outputs within journal articles) was approximately four times less common.

### Facilitating different types of reuse

**Fig. 3:**
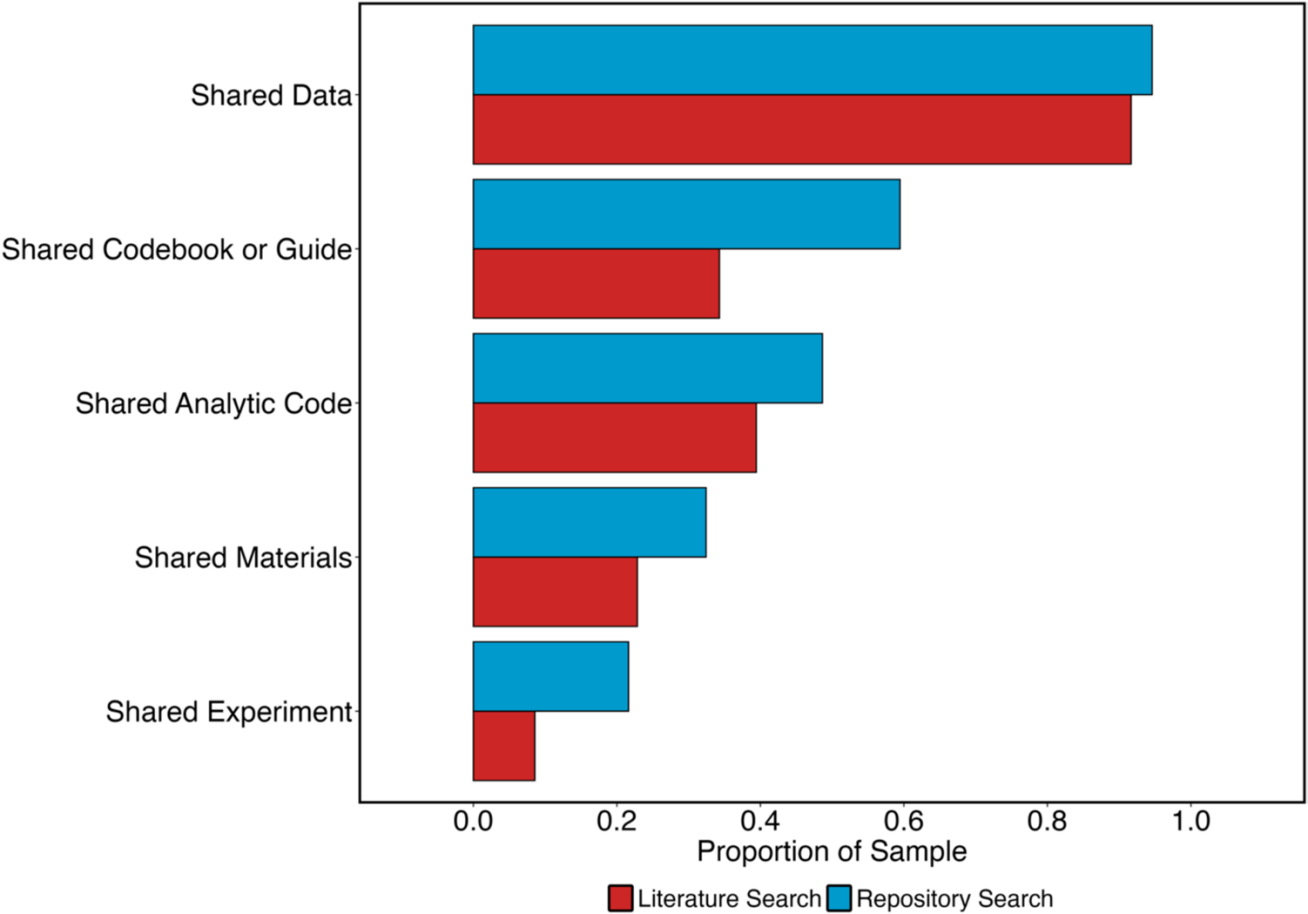
Bar chart showing rates of output sharing for different types of outputs from our literature search and repository search. *Note.* See main text for definitions of terms

Next, we examined the outputs that had been shared in terms of whether they could facilitate different types of reuse, summarized in Fig. 3.

#### Supporting analytic reproducibility

We first assessed the outputs to determine their analytic reproducibility. We assessed this by cataloguing whether researchers shared datasets and/or relevant analytic code.

For both of our searches, researchers commonly shared some form of dataset (91.67% for our literature search and 94.6% for our repository search). However, considerably fewer researchers shared analytic code, despite our use of a liberal criterion to determine whether code was shared. We recorded an affirmative response if researchers shared any form of code, not checking whether the code could compile or run whether it could reproduce the results of the study. For our literature search, 39.4% (*N* = 28) shared code, compared to 48.6% (*N* = 18) for the repository search.

We also examined the file formats in which the code was shared. Here, we coded the file formats as ‘open’ when they were made using open-source frameworks, such as Python and R. Sharing code in closed formats can make it difficult for others to run code and reproduce findings. We coded file formats as ‘closed’ when they were made using proprietary formats such as MATLAB and Excel. In some cases, researchers shared analytic code in both open and closed formats. In our literature search, 78.57% of publications (*N* = 22) shared analytic code in open formats, compared with 88.89% of outputs (*N* = 16) in our repository search. Likewise, 25% of publications (*N* = 7) from our literature search shared code in closed formats, compared with 11.11% of outputs (*N* = 2) from our repository search.

To summarize, researchers commonly shared datasets and less commonly shared analytic code. Generally, when sharing code, researchers use open formats. The high prevalence of datasets and low rates of sharing metadata (see below) being shared suggests that, when researchers in our field share outputs, they primarily consider sharing to be for the purpose of analytic reproducibility, although the ability of others to reproduce analyses was limited because sharing of code was less common in both of our searches.

#### Supporting direct replications

Next, we assessed whether shared outputs could facilitate *direct replications*. Replication can be supported by sharing experimental software code and other materials (e.g., images, videos, sounds) from experiments. A secondary benefit of sharing code is that in the absence of a codebook, the experimental software itself can aid in understanding datasets and their variables/columns.

The rate of sharing experimental software or code was very low for both searches. For our literature search, 8.6% (*N* = 6) of the sample shared experimental software and code, compared with a higher rate of 21.6% (*N* = 8) of the sample from the repository search. The rate of sharing materials was similarly low: For our literature search, 22.86% (*N* = 16) shared materials; for our repository search, 32.43% (*N* = 12) shared materials.

How openly accessible were the experiments that were shared? It is important to address the accessibility of the shared experiments because sharing experiments using proprietary software can pose a barrier to other researchers. In our literature search, two of the six experiments shared were in an open format (one using PsychoPy and one using Open Sesame), while four were shared in a closed format (two using MATLAB, two using E-Prime). For our repository search, three of the eight experiments were shared in an open format (Open Sesame), while five were shared in a closed format (three using E-Prime, two using MATLAB).

Because the rate of experimental software or code sharing was so low across both searches, it is difficult to draw concrete conclusions regarding experiments that are openly accessible for use by other researchers. However, most experiments were shared in a closed format, limiting the ability of other researchers to reuse shared outputs. Unlike analytic reproducibility, it appears that researchers in our field do not often seek to facilitate direct replications of their work when deciding what to share.

#### Facilitating new research via secondary data analyses

As noted above, the most common form of output shared by researchers in both searches consisted of experimental data. Next, we examined whether these shared datasets could facilitate new research via secondary data analyses. For this to happen, ideally, researchers will share raw rather than final datasets.

The ideal raw data to facilitate new research are *by-fixation* datasets, which break data into one row per fixation conducted and do not average across trials or participants. These break the data down with one row per fixation in the experiment and do not average across trials or participants.^1^ Because of this, researchers may not typically consider sharing by-fixation data if they instead focus on sharing the datasets necessary to analytically reproduce the results of their experiments.

By-fixation datasets can be averaged in different ways to produce the final dataset for analysis. Researchers can summarize by both participant and trial to create *by-trial* datasets (e.g., the participant’s average fixation duration per trial); they can then take these by-trial datasets and average across trials by participant IDs to create *by-participant* datasets (e.g., the participant’s average fixation duration across all trials). By-participant analyses lend themselves to standard statistical tests such as *t* tests and ANOVAs and thus are the data in which those seeking to analytically reproduce a study are most likely to be interested. To take an example, suppose a researcher is interested in examining mean fixation counts using an ANOVA. First, they must calculate the average number of fixations for each participant and each trial. This creates a by-trial dataset where each row comprises the average number of fixations for each trial of each participant. Next, the researcher must take the average number of fixations across all trials to create the average number of fixations for each participant. If the by-trial step is not taken, the final by-participant means can be inaccurate (Godwin et al., 2021).

**Fig. 4:**
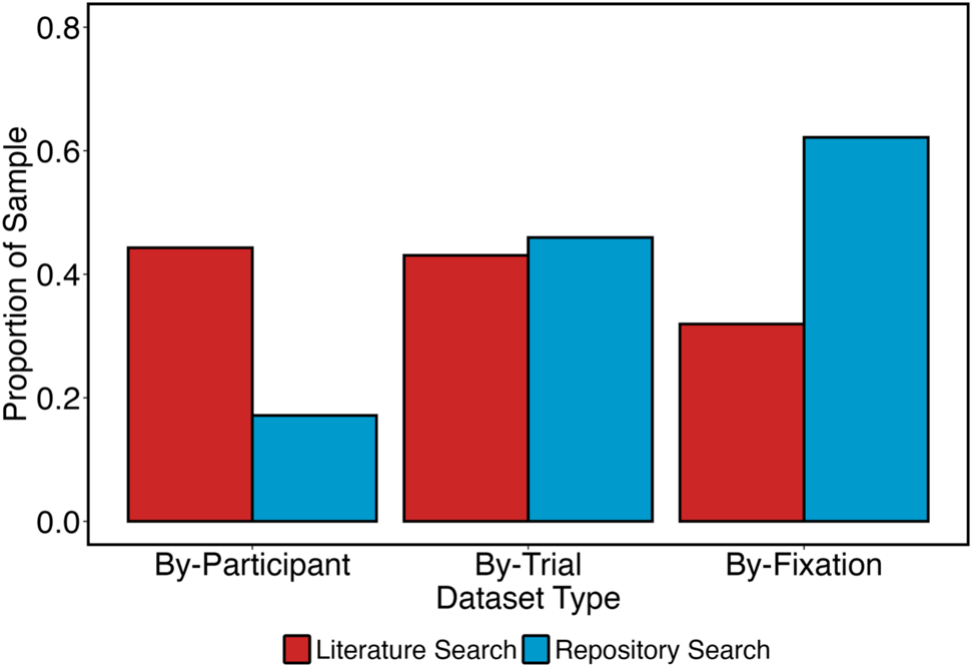
Rates of sharing different types of datasets from our literature search and repository search

We therefore examined the rate at which researchers shared by-fixation data (i.e., raw data), by-trial data, and finally by-participant data. We present descriptive statistics in relation to these in Fig. 4. Although the overall rate of sharing data was similar between the two searches (as can be seen in Fig. 3), the *types of data shared* was often very different from the results returned by the two searches. There was a higher rate of sharing of by-fixation datasets in our repository search than our literature search, and this finding was reversed for by-participant datasets. The rate of sharing by-trial datasets was similar for both searches.

Finally, we examined whether the datasets shared by researchers were shared in a closed versus open format. Our assessment of this issue was made more complex by the fact that, in some cases, researchers shared the same dataset in different formats, including both open and closed formats. For that reason, we coded what was shared using a simplified approach, charting the proportion of publications that shared datasets in a closed format, as well as the proportion of publications that shared datasets in an open format. Because the same datasets were often shared in multiple formats, many publications received an affirmative response on both criteria. We coded as ‘closed’ proprietary data files, such as those from experiments that used MATLAB, those that used SR Research Experiment Builder, and Excel and Word files. Raw text files and those files that can be opened readily by open-source tools such as R and Python were coded as ‘open’.

**Fig. 5:**
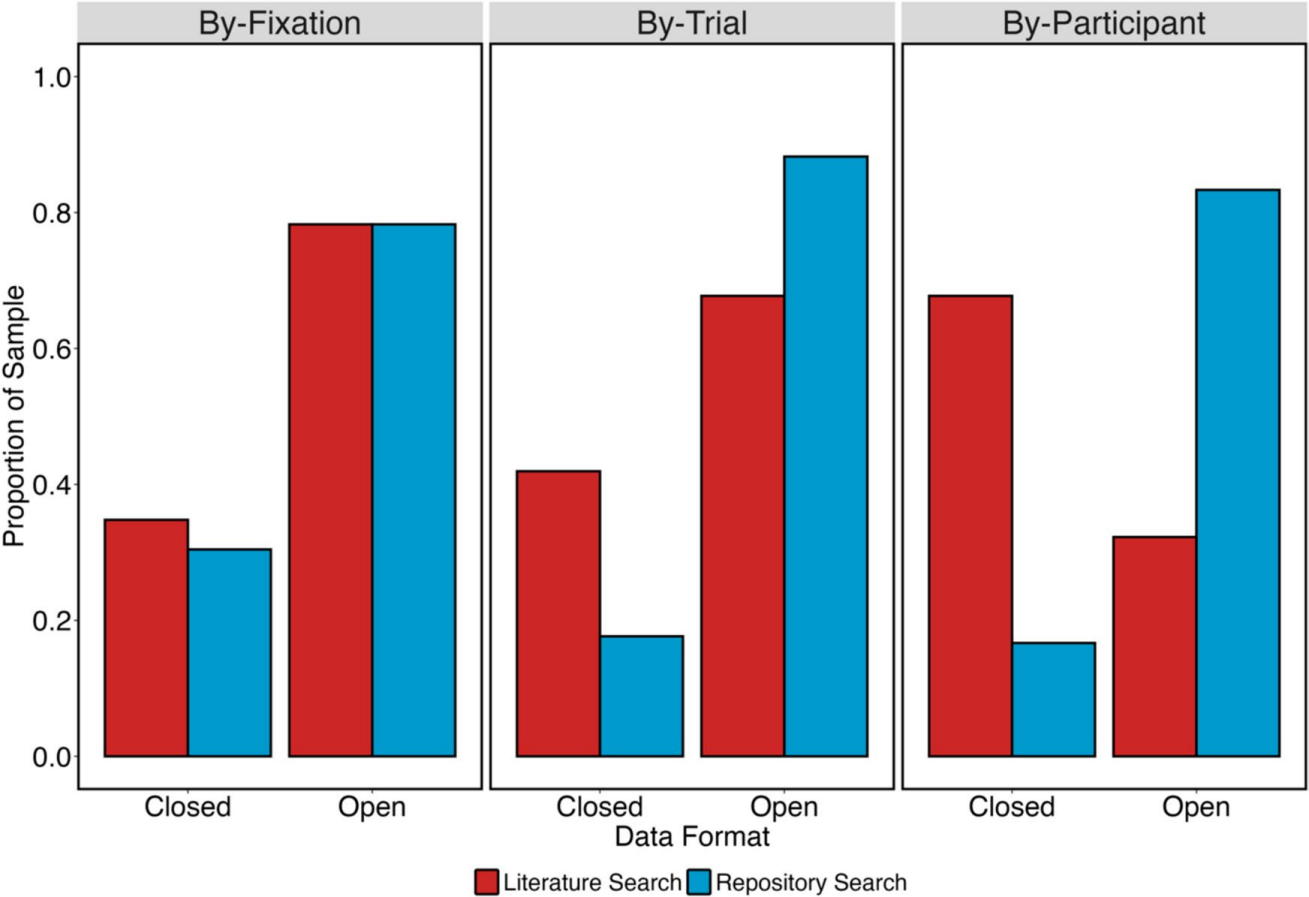
Proportion of samples sharing by-fixation, by-trial and by-participant datasets in open and closed formats

We present the results of this examination in Fig. 5. For both the literature search and the repository search, by-fixation datasets were primarily shared in an open format, and this was true for by-trial datasets as well. For our literature search, by-participant datasets were primarily shared in a closed format but were primarily shared in an open format for our repository search.

### Facilitating reuse with metadata

Without metadata, codebook, guide, or other information describing shared outputs, it can be difficult to understand what those outputs are, whether they are trustworthy, and/or how to reuse them. Rich and accurate metadata is important for all types of reuse. To evaluate metadata sharing by researchers in our samples, we asked: *Did researchers share a codebook or guide for their outputs?* We used a liberal criterion for responding ‘yes’, namely whether researchers shared even a simple codebook describing the dataset(s). In our literature search, 34.3% (*N* = 24) of the sample shared guides, while a higher rate of 59.45% (*N* = 22) did so in our repository search.

### Comparing our two searches

Since we conducted two searches with two different starting points, this offered us a novel opportunity to study how findable the outputs were. As the findings from our literature search were narrowed down to just those shared on the OSF, theoretically, we should have found *more projects* on the *OSF* during our repository search than during our literature search. This is because our repository search was not limited to specific journals (since doing so was not possible), unlike our literature search. Instead, we found the two searches largely found *different OSF projects*. There were 71 OSF projects found by at least one of the searches. Of these, only nine (~13 %) were found by both searches, 34 (~48%) were found only via our literature search, and 28 (~39%) were found only via our repository search.

Why did the two searches find largely non-overlapping sets of studies? The answer to this lies in the metadata upon which the searches were based. Metadata can take many forms, including guidebooks or data dictionaries, or any other additional information supplied, including associated publication titles, abstracts, and publication outlets. The literature search scrutinized publication titles and abstracts, whereas the repository search scrutinized project titles, descriptions, and other sources of information available in each repository. The difference between search results is due to publications *requiring* authors to include titles and abstracts that are meaningful, accurate, and relevant, whereas OSF projects do not have such requirements. Indeed, OSF projects can be almost entirely lacking in searchable metadata.

**Fig. 6:**
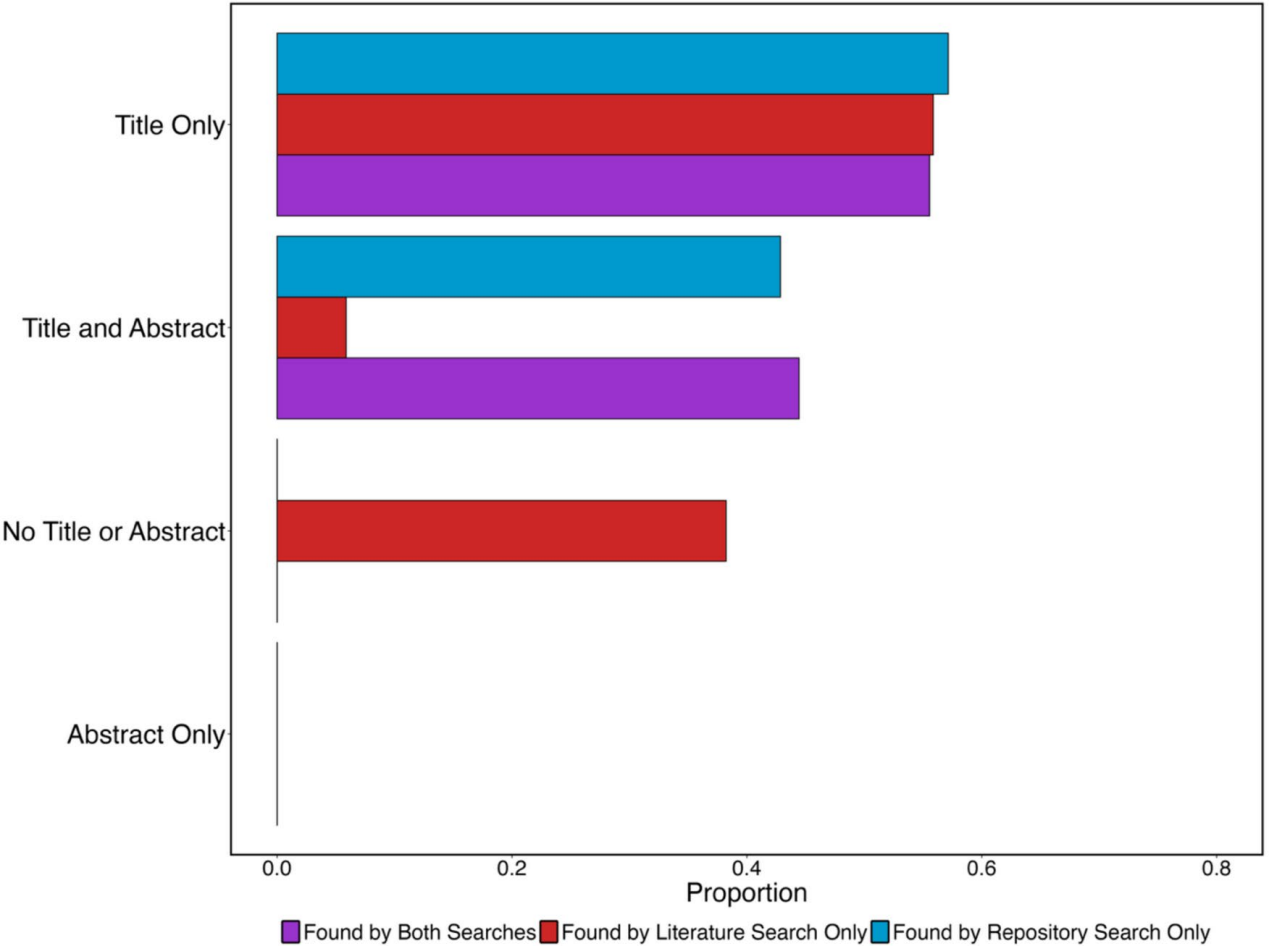
Bar chart showing the proportion of OSF Projects returned by our two searches that included different types of metadata in their project information

To capture this divergence between the two searches in detail, we examined the OSF projects from our two searches and checked whether they included the titles and abstracts of their associated publications. This involved asking the question: *Were article titles and abstracts included in OSF project metadata?* As can be seen in Fig. 6, there were three general approaches taken by researchers regarding this question. First, only about half of the projects included the titles of articles in their metadata. Second, just under half of the projects identified by our literature search lacked either a title or an abstract in their metadata. Finally, just under half of the projects found by our repository search only included both titles and abstracts. The fact that projects identified only through our literature search and those identified only through our repository search employed different approaches to metadata can explain why there was so little overlap in the projects found by our two searches. Overall, then, when researchers shared both titles and abstracts from associated publications in OSF project metadata, this substantially increased the likelihood that those projects will be found when a repository search is conducted.

## Discussion

To fulfil the promise of Open Science, academic stakeholders have become increasingly interested in characterizing the research output sharing practices of researchers, both across disciplines and subfields (e.g., see Hardwicke et al., 2020, 2022, 2024; Hardwicke & Ioannidis, 2018; Roche et al., 2015; Towse et al., 2021), and within specific journals and sub-fields (e.g., Crüwell et al., 2023; Hardwicke et al., 2018; Huff & Bongartz, 2023; Laurinavichyute et al., 2022; Rochios & Richmond, 2024; Wiechert et al., 2024). We built on this research by conducting what we refer to as a sharing practices review of outputs from publications in our field of visual search and eye movement behavior, using two searches: a literature search and a repository search. We catalogued the outputs according to the types of reusability they could facilitate, including analytic reproducibility, direct replications, and secondary data analyses.

The most common practice adopted by researchers in our literature search was to share no outputs. Of those who did share outputs, in both searches, researchers primarily shared datasets. Unfortunately, the sharing of metadata such as codebooks, data dictionaries or guides was rare as was the sharing of analytic code alongside datasets. Most of the analytic code shared was done so in a closed format, limiting the ability of other researchers to reproduce analyses.

Researchers rarely shared outputs that could support direct replications. The sharing of experimental code and materials was sparse, a trend observed in both the outputs examined as part of our literature search and repository search. When code was shared, it was primarily shared in a closed format, using code from MATLAB experiments. This further limits the ability of the outputs to support direct replications, as they require the use of proprietary software that necessitates specific licenses. Unlike sharing for the purposes of analytic reproducibility, researchers in our field do not generally appear to consider sharing for the purposes of direct replications.

There was a divergence in our searches in terms of their ability to support secondary data analyses. The ideal data format for a secondary data analysis is raw experimental data. In the context of visual search and eye movement experiments, the raw data that we sought consisted of by-fixation datasets. These datasets were shared more frequently by publications found through our repository search, and seldom by publications through our literature search. Shared raw by-fixation datasets were primarily shared in an open format. This divergence highlights variability in the approaches taken by researchers, with some sharing raw data that is valuable for secondary data analyses, and others not. Of course, it is possible that the sharing of raw data in these cases was not motivated by the facilitation of secondary data analyses. Instead, by-fixation datasets may have been shared by researchers whose analyses focused on those datasets without summarizing them in other forms, such as by-participant datasets. Nevertheless, we note that some researchers share outputs that can facilitate the creation of secondary datasets at a considerable rate.

Taken together, these patterns of sharing that can support different types of reuse lead us to conclude that researchers in our field primarily consider the act of output sharing as being one that is focused on facilitating analytic reproducibility. Across all outputs shared, the act of understanding and determining what was shared was a constant challenge during this project because the sharing of metadata was so limited. We often found ourselves scouring datasets with no information regarding what was contained within them. Dataset names often gave few clues regarding what was contained within, and column names within those datasets could often be cryptic, typically involving combinations of a small number of letters that clearly had some meaning to the authors but were virtually impossible for outsiders to understand. Many data file names were likely generated by software during data collection, and therefore, their contents and meaning were not readily apparent. Overall, it seemed to us that there was a consistent problem in that researchers might share outputs under the assumption that ‘it makes sense to me,’ without considering how difficult it would be for others to comprehend what had been shared.

When seeking to find outputs for the purposes of analytic reproducibility, direct replications, or secondary data analyses, what do our examinations suggest is the best form of search to use: a literature search or a repository search? Overall, sharing in support of all forms of reproducibility was higher in our repository search than our literature search, so the success rate of finding viable outputs is likely to be higher when searching for outputs using an online repository such as the OSF. However, this suggestion must be tempered by the fact that the overall number of results returned by our repository search was far lower than for our literature search.

Of course, this situation does not need to persist. We noted in our cross-comparison of the results of both searches that our repository search missed many viable projects and outputs because projects were not shared with sufficient metadata to enable them to be found. Many projects identified through our literature search lacked relevant information regarding the associated publications, including publication titles and abstracts. Including these titles and abstracts increases the likelihood that projects will be found by researchers when searching a repository.

If researchers were to include titles and abstracts in these projects, a future repository search would likely find them. Doing so will enable future searchers seeking valuable outputs to avoid a literature search entirely. Literature searches are laborious and inefficient, involving the examination of titles and abstracts of potentially relevant publications, and then reading those publications to identify connections to relevant outputs. Links to outputs are rarely placed at consistent locations across publications, even within the same journal, making it difficult to find those links or confirm their absence. Moreover, many links do not function at all. A repository search has the potential to be more efficient by avoiding these steps and taking a searcher directly to the outputs: all that is needed is for those outputs to be more *findable*, per the FAIR principles.

A key question that emerges from our findings is why sharing practices are so variable and so sparse in this field? Several surveys have highlighted barriers to sharing outputs, including datasets. Houtkoop et al. (2018) found that researchers reported reluctance to share datasets because they perceive a lack of norms within that their about sharing datasets, and that they lack the time and appropriate training to share datasets. They also found that researchers lacked incentives to share, and encountered ‘fear-based’ barriers, such as other researchers finding errors within their shared datasets. Uncertainty about appropriate practices also hinders the sharing of sensitive or identifiable data from human subjects (Washburn et al., 2018). Given these barriers, it is perhaps not particularly surprising that the outputs shared here were, in many cases, quite rare.

Past research has provided guidance and recommendations to improve output sharing practices. As with the FAIR principles (Wilkinson et al., 2016), which have been criticized for their generality (Candela et al., 2024) and lack of clear actionable behaviors for active researchers to engage in (Henriksen & Mundt, 2024). Without appropriate specificity, incentives, or mandates, recommendations and guidance may be insufficient in improving researchers’ output sharing. We believe that providing very specific guidance and templates would be more helpful in supporting better sharing practices, thereby saving researchers’ time and promoting consistent output sharing across researchers, teams, and fields. Most researchers have little time to take on new activities or learn, for example, the minutiae of what is required to comply with the FAIR principles. With that in mind, we have encapsulated our recommendations in a template, described below. By providing something easily understandable and readily usable, we hope to promote at least the accurate cataloging of sharing practices, and, ideally, to prompt researchers to realize that sharing is not just to facilitate analytic reproducibility.

### The Find It, Access It, Reuse It scorecard

Drawing on our findings, we have developed the *Find It, Access It, Reuse It* scorecard, listed in Table 1, written for researchers both within and beyond the eye-tracking community:

1. *Researchers* can access an online copy of the scorecard in text form via our OSF project for this publication, fill it in, and then copy the text to online repositories where they have shared outputs. Using the scorecard will prompt researchers to consider and enhance the findability, accessibility, and prospects for different forms of reuse of their outputs. We have included basic text here in italics to highlight how to answer each scorecard question.
2. *Journals, funders, and other high-level organizations* could, for example, require that researchers complete this scorecard (or some variant of it) when sharing their outputs alongside publications.
3. *Online repositories* could embed this scorecard into their existing templates, prompting researchers to complete this scorecard when sharing their work outputs, and then automatically incorporating it into a repository or project. Online repositories could also incorporate information from scorecards into search filters, for instance, the OSF could include a filter to select only the projects that have been marked by researchers as containing outputs for direct replication. This approach could vastly improve the process of searching for existing outputs, and reduce the time and effort spent examining projects containing outputs not relevant to the searcher.
4. *Other researchers* can use these scorecards when conducting research in combination with software to poll, *en masse*, the contents of outputs shared by researchers online.

**Table 1:** *Find It, Access It, Reuse It* scorecard for promoting findability, accessibility, and reusability of scientific outputs. Please Note: This is not intended as a ‘judgmental’ checklist. Not all queries need to be answered in the affirmative. The goal is description (what is there?) and encouragement (what else might make sense to add?)

Category	Response *User notes in italics.*
**Find It: Making shared outputs more findable online** *Completing this section will help others to find your work more easily*	
Associated Publication Title	*Make sure to set your project or repository title to match that of the publication and then paste that title here. Including the publication title makes it easier for others to find the shared outputs.*
Associated Publication Abstract	*Include your abstract from your paper as part of your project or repository description/metadata and then paste that abstract here. Including the abstract makes it easier for others to find the shared outputs.*
Associated Publication Authors	*List authors as part of your project or repository description/metadata and then paste them here as well.*
Associated Publication Journal	*Make sure to include the journal name as part of your project or repository description/metadata and then paste it here as well.*
DOI of Associated Publication	*Include the DOI of your publication in the project or repository description/metadata, and after that paste it here.*
Associated Publication Status	*Published/Under Review/etc.*
Publication Year	*Write ‘N/A’ if not published or enter year of publication here.*
**Access It: Checking that outputs can be accessed** *Completing this section will make sure that your work can be accessed*	
Where have the scientific outputs been shared online?	*Paste web address here.*
Has the web link to the outputs been checked?	*Could a reader gain access? This should be checked by someone not connected with the creation of the archive. When checking, we encourage the use of ‘Incognito’ or ‘Private’ browsing modes to ensure the outputs are not just available to logged-in users.*
**Reuse It: Understanding how the outputs are reusable** *Completing this section will help others to understand what you have shared, and for what it can be used. Outputs can be reused for many different purposes and here we prompt you to consider them. For any ‘yes’ responses, write in brackets after the word ‘yes’ a comma-separated list of names of any files and folders that are relevant*	
Has a guide to the outputs been included?	*This guide can simply consist of a list telling the reader the contents of each shared folder/file and for what purpose it is used.*
Has a guide for any shared datasets been included?	*Ideally, this would include a list explaining what each column in each shared dataset is presenting.*
Has the raw data been shared?	*Add the file name(s) here, or ‘No’ if not shared.*
Has the code for running the experiment been shared?	*Add the file name(s) here, or ‘No’ if not shared.*
Have you shared the results of analyses that were conducted?	*Add the file name(s) here, or ‘No’ if not shared.*
Have you shared the code for deriving those results from the data?	*Add the file name(s) here, or ‘No’ if not shared.*
Have all outputs been shared in an openly accessible format where possible?	*Consider sharing data outputs as raw text files to enable others to gain easier/clearer access to those outputs.*

Note: This version is written in a format that enables readability in a publication format

We recommend that the scorecard be used to catalogue shared outputs in a highly visible manner to others. Our recommendation is that the scorecard, once filled out, be pasted into any general description or introductory information relating to shared outputs. To show how this might be implemented in practice, we have set up a series of examples. First, we have created example projects in popular repositories, having filled out the scorecard for the current publication. These examples include the OSF (https://osf.io/5tmey/), Zenodo (https://zenodo.org/records/15731333), and Figshare (https://figshare.com/projects/A_Sharing_Practices_Review_of_the_Visual_Search_and_Eye_Movements_Literature_Reveals_Recommendations_for_our_Field_and_Others/253979). Second, we have created an example scorecard for a separate publication (Dewis et al., 2025) and uploaded that as well (available at: https://osf.io/2zyvf/).

The *Find It, Access It, Reuse It* scorecard is structured in the following manner, drawing upon the basic terminology of the FAIR principles and infusing that terminology with the findings from our two searches here:

- ***Find It:* Making shared outputs more findable online:** In this section, basic information regarding the outputs’ associated publication is presented. We have included this because many of the samples that we examined in both searches did not make this information readily available. Including information such as titles and abstracts will not only aid those who have found outputs via a repository search find the associated publication but will also make it more likely that outputs will be found when relevant search terms are used.
- ***Access It:* Checking that outputs can be accessed:** In this second section, we prompt researchers to list where the outputs are shared and to check that the outputs can be accessed by everyone. This includes a recommendation to check the link to the outputs to ensure that they are open and not only available to users who are logged in to a particular website or repository.
- ***Reuse It:* Understanding how the outputs are reusable:** In this final section, we prompt researchers to consider and catalogue what different forms of reuse are facilitated by the outputs that they have shared. These include analytic reproducibility, direct replications, and secondary data analyses. Because all of these forms of reuse are difficult – if not impossible – without adequate metadata, in this section we also suggest that researchers consider sharing a codebook/guide relating to their outputs.

## Conclusions and future work

Our two searches (and comparisons between them) have revealed considerable scope for improving data sharing practices and how easily outputs can be found, in visual search and eye movement research. Repeating this process for different fields and subfields would be useful to see if our results generalize elsewhere. Platforms like the Open Science Framework might encourage changes in reporting that would make searching more successful. We plan to follow up by contacting researchers whose outputs were found exclusively by our literature search and suggesting that they improve their outputs’ findability by augmenting metadata, such as by adding publication titles and access, ideally by using our *Find It, Access It, Reuse It* scorecard. We can then repeat the repository search to assess the impact of whether our efforts have enhanced these projects’ findability.

Finally, the results of our two searches have a useful by-product: our cataloguing of outputs can be used as a database to enable others in our field (including ourselves) to more readily find outputs of value to them. We have made this available online (via this publication's shared outputs: https://osf.io/5tmey/) and plan to periodically update this database, ideally by working with others in our field to identify new additions or modifications in the future.

## Footnotes

1 Technically, there is a ‘rawer’ form of data than by-fixation data that comprises individual samples recorded by the eye tracker during a study. For instance, an eye tracker that records at 1000 Hz (i.e., 1000 samples per second), would produce an impractically large dataset comprising tens of millions of rows of data even for a simple study. Consequently, we treat by-fixation datasets as the ideal raw data for secondary data analyses.
